# Supplementary material for: Stabilized ε-Fe2C catalyst with Mn tuning to suppress C1 byproduct selectivity for high-temperature olefin synthesis
Source: Nat Commun. 2024 Jun 15;15:5128. doi: 10.1038/s41467-024-49472-x (PMC11180106; doi:10.1038/s41467-024-49472-x)
Supplement: Supplementary file 1 — Supplementary Information [file 41467_2024_49472_MOESM1_ESM.pdf]

Supplementary Information

Stabilized ε-Fe_2C Catalyst with Mn Tunning to Suppress C1 Byproduct Selectivity for High-Temperature Olefin Synthesis

Fei Qian[1,2,3,†], Jiawei Bai[1,2,3,†], Yi Cai[1,2,3,†], Hui Yang[1,2,3], Xue-Min Cao[1,2,3], Xingchen Liu[1]*, Xing-Wu Liu[2]*, Yong Yang[1,2,3], Yong-Wang Li[1,2,3], Ding Ma[4], Xiao-Dong Wen[1,2,3]*

[1]State Key Laboratory of Coal Conversion, Institute of Coal Chemistry, Chinese Academy of Sciences, Taiyuan, 030001, China; [2]National Energy Center for Coal to Liquids, Synfuels China Co., Ltd., Huairou District, Beijing, 101400, China; [3]University of Chinese Academy of Sciences, No. 19A Yuquan Road, Beijing, 100049, PR China; [4]Beijing National Laboratory for Molecular Sciences, New Cornerstone Science Laboratory, College of Chemistry and Molecular Engineering, Peking University, Beijing, China.

[†] These authors contributed equally: Fei Qian, Jiawei Bai, Yi Cai.

* Email: liuxingchen@sxicc.ac.cn; liuxingwu@sxicc.ac.cn; wxd@sxicc.ac.cn

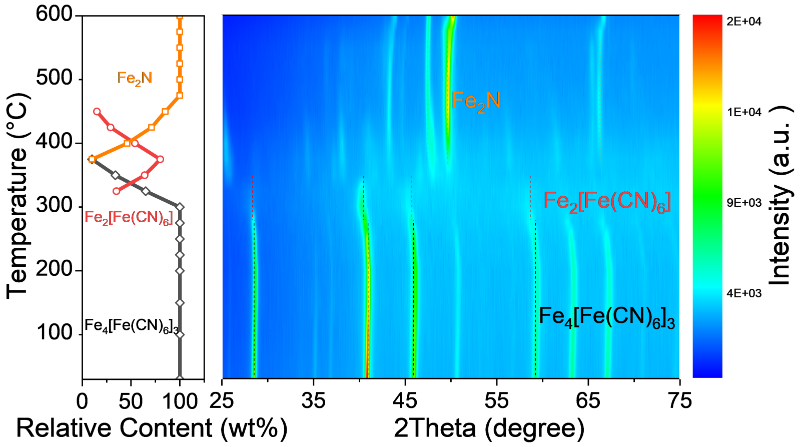

Supplementary Fig. 1 | In situ XRD patterns. Pyrolysis process in NH_3 for Prussian blue. Pyrolysis conditions: 50 mL·min^{-1} NH_3, 5 °C·min^{-1}.

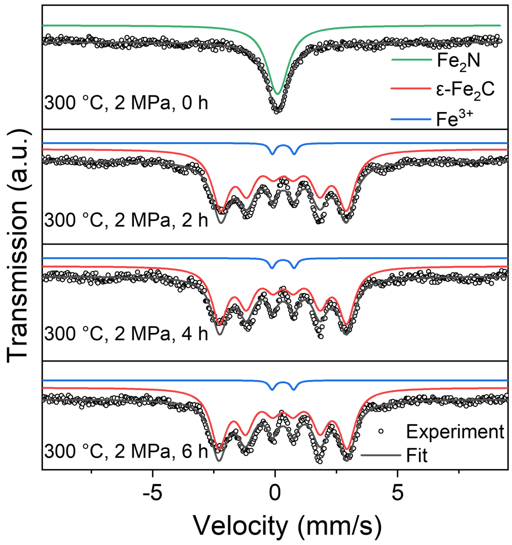

Supplementary Fig. 2 | Mössbauer spectra of ε-Fe_2C-0.4Mn catalyst. The **ε-**Fe_2C-0.4Mn firstly pre-treated for 1h at the condition of 550 °C, in 100% ammonia gas. The sample was subsequently exposed to the condition of 300 °C, GHSV= 20 L·g_{cat}^{-1}·h^{-1}, 2 MPa, H_2/CO ratio of 2.5 and kept for 0 h, 2 h, 4 h and 6 h respectively. The Mössbauer spectra data was acquired in the 10 K.

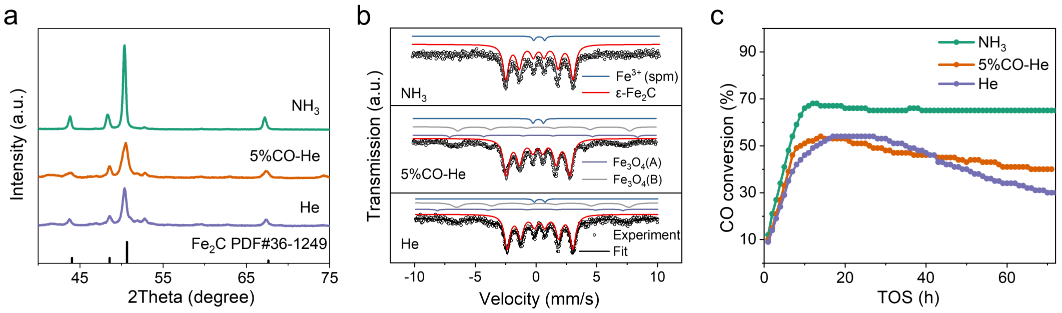

Supplementary Fig. 3 | Structure and catalytic performance of ε-Fe_2C (after FTS) catalyst under different activation conditions. **(a)** XRD, **(b)** MES and (**c**) catalytic performance of ε-Fe_2C catalyst under different activation conditions. Reaction conditions: 0.10 g catalyst, 270 °C, GHSV= 60 L·g_{cat}^{-1}·h^{-1}, 2 MPa and H_2/CO ratio of 2.

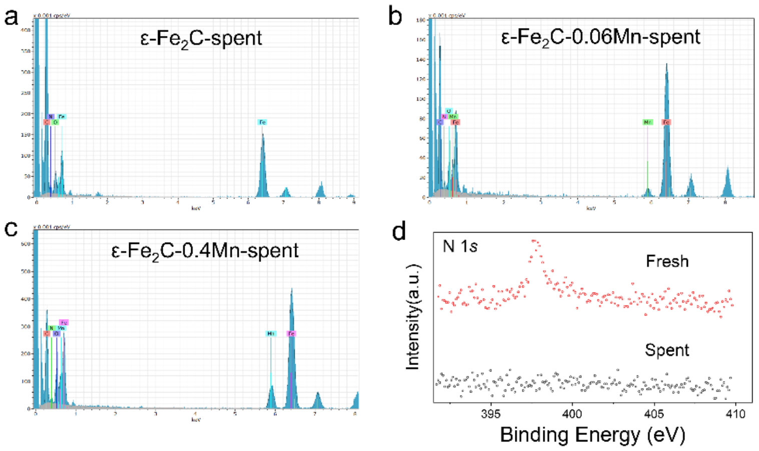

Supplementary Fig. 4 | (a, b, and c) X-ray energy dispersive spectroscopy image of ε-Fe_2C-xMn (x = 0, 0.06, 0.4). (d) XPS profiles in the N 1*s* of ε-Fe_2C before and after FTS reaction.

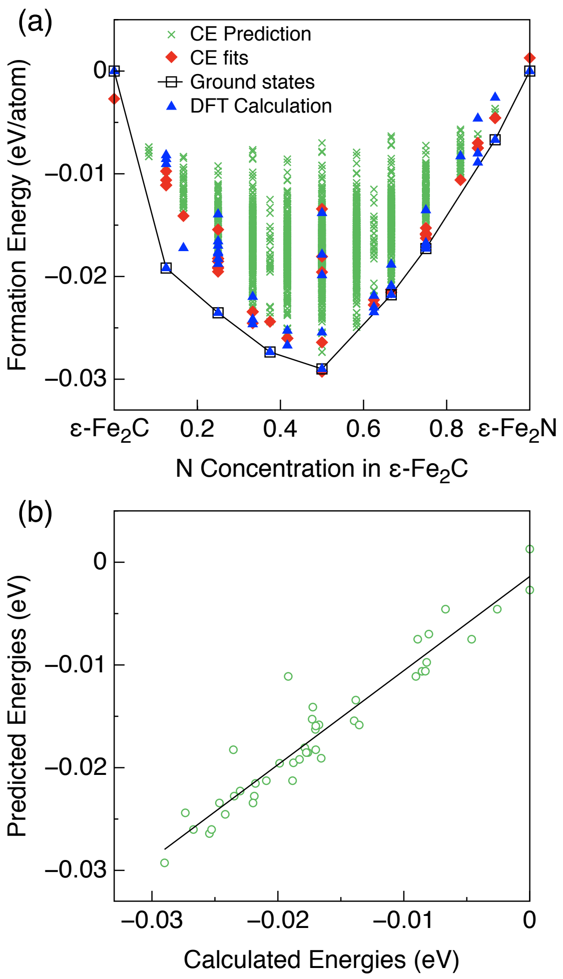

Supplementary Fig. 5 | (a) Formation energies predicted by Cluster Expansion method for 2868 different N configurations. (b) The relationship between predicted energies and DFT calculation energies.

We utilized the Automated Toolkit for Atomistic Simulations (ATAT) in conjunction with the Vienna Ab initio Simulation Package (VASP) to calculate the formation energy of nitrogen-doped ε-Fe_2C. Initially, potential doping structures were predicted using the Monte Carlo-based mcsqs program from ATAT. Subsequently, the maps program from ATAT was employed to generate supercells based on these predicted structures. DFT calculations were then performed using VASP, with appropriate exchange-correlation functionals and pseudopotentials. The formation energy was calculated from these DFT results, taking into account the total energy of the doped structure, the energy of pure Fe_2C and pure Fe_2N.

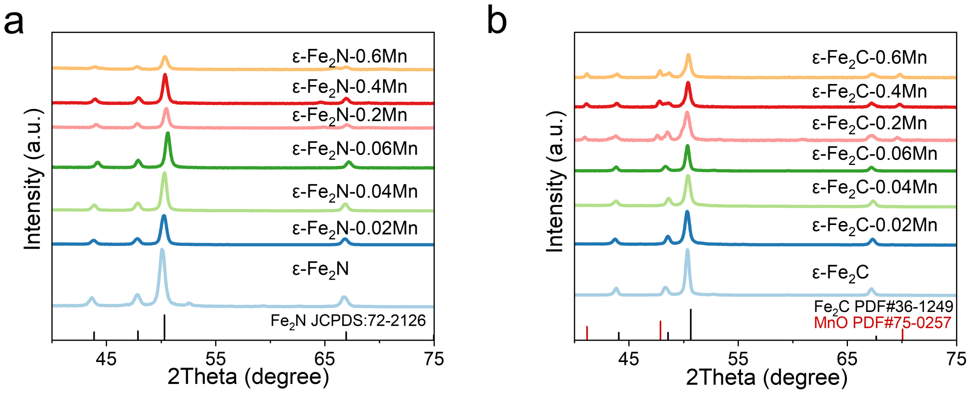

Supplementary Fig. 6 | XRD characterization. XRD patterns of (a) ε-Fe_2N (fresh) and (b) ε-Fe_2C (after FTS) with different manganese additions level.

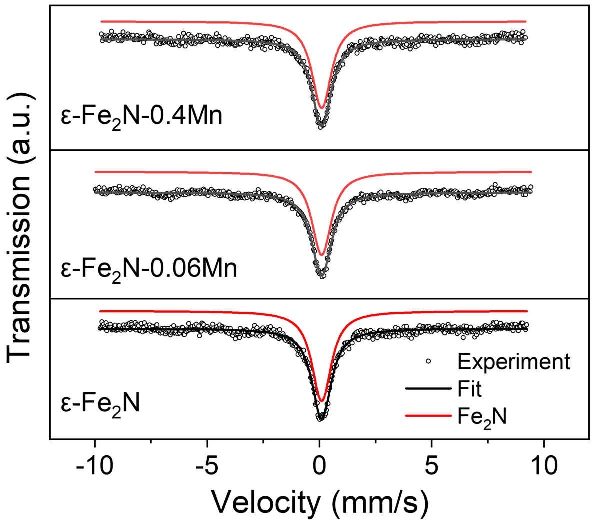

Supplementary Fig. 7 | Mössbauer spectra of ε-Fe_2N catalysts with different manganese additions before reaction.

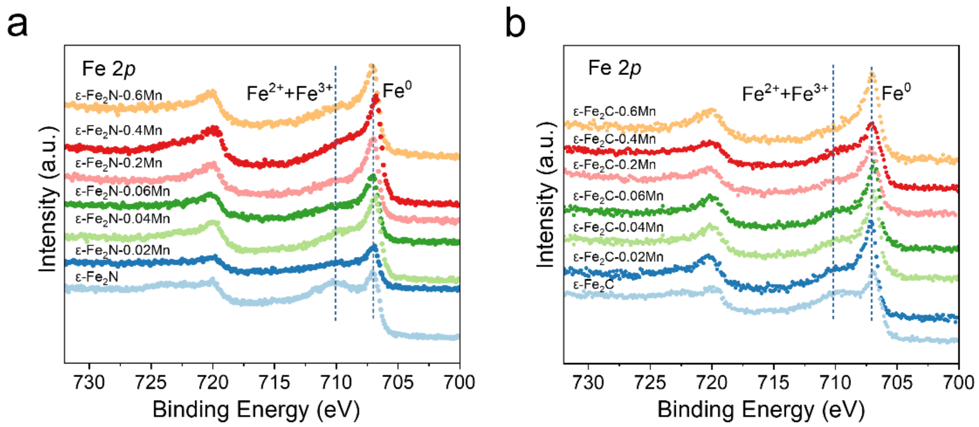

Supplementary Fig. 8 | XPS profiles in the Fe 2*p* for **(a)** fresh and spent **(b)** ε-Fe_2C-xMn (x= 0, 0.02, 0.04, 0.06, 0.2, 0.4, and 0.6) catalysts.

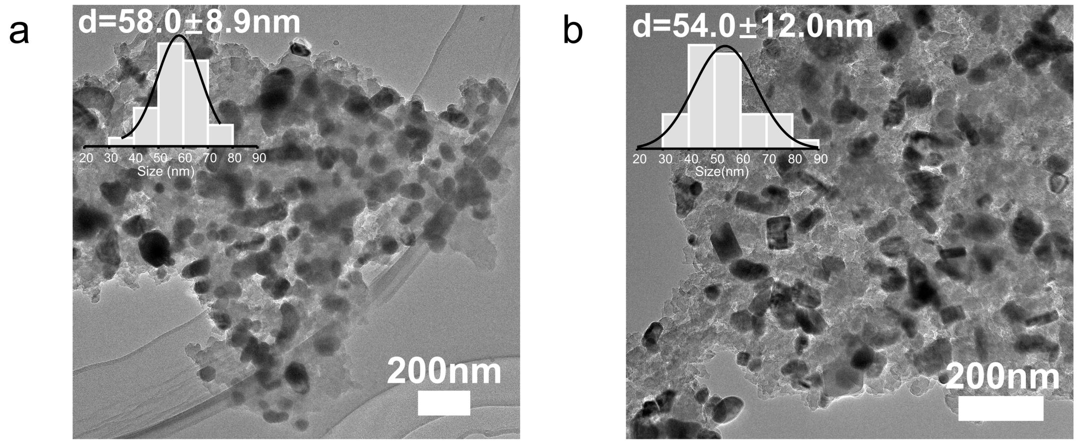

Supplementary Fig. 9 | TEM images and size distribution. (a) ε-Fe_2C catalyst **(b)** ε-Fe_2C-0.06 Mn catalysts.

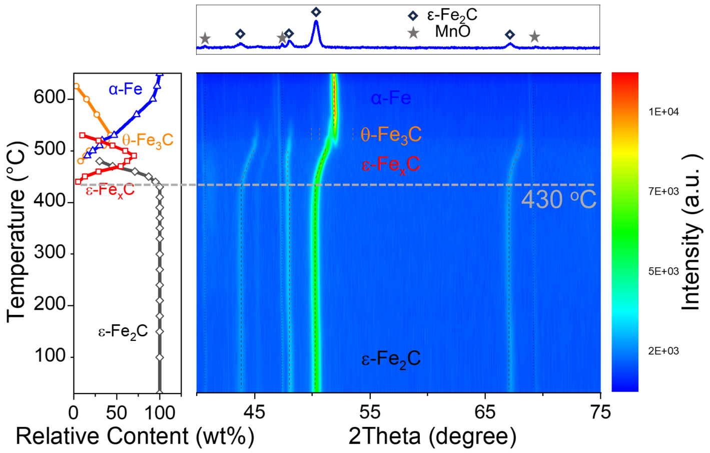

Supplementary Fig. 10 | *In situ* XRD patterns of the temperature-programmed phase transition of two ε-Fe_2C-0.4Mn samples in a helium atmosphere from room temperature to 650 °C. Conditions: He 50 mL·min^{-1}, 5 °C·min^{-1}. The catalysts were activated from Prussian blue analogs and reacted for 50 h under the reaction conditions, followed by switching to a He flow, then the catalysts were measured by XRD as the temperature changed.

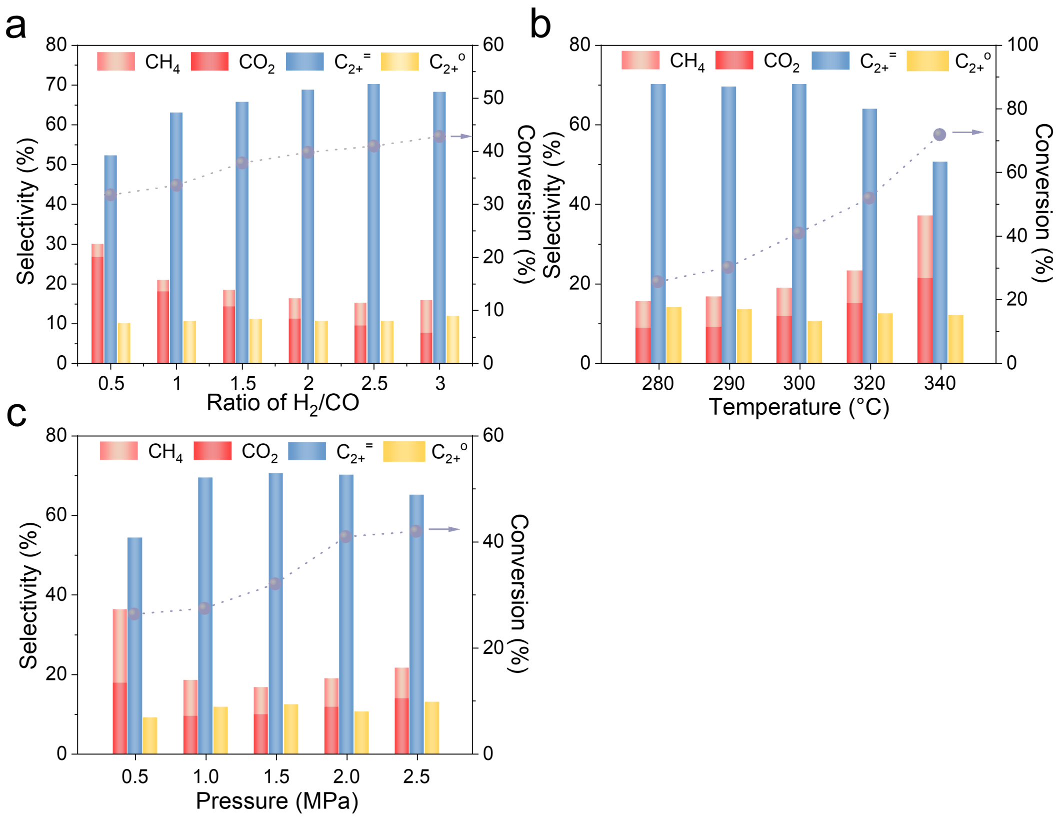

Supplementary Fig. 11 | Catalytic Performance. **(a)** Conversion of CO and product selectivity of ε-Fe_2C with different ratio of H_2/CO. Reaction conditions: 0.10 g catalyst, GHSV= 20 L·g_{cat}^{-1}·h^{-1}, 2 MPa and temperature of 300 °C. **(b)** Conversion of CO and product selectivity of ε-Fe_2C with different temperature. Reaction conditions: 0.10 g catalyst, GHSV= 20 L·g_{cat}^{-1}·h^{-1}, 2 MPa and H_2/CO ratio of 2.5. **(c)** Conversion of CO and product selectivity of ε-Fe_2C with different pressure. Reaction conditions: 0.10 g catalyst, GHSV= 20 L·g_{cat}^{-1}·h^{-1}, 300 °C and H_2/CO ratio of 2.5.

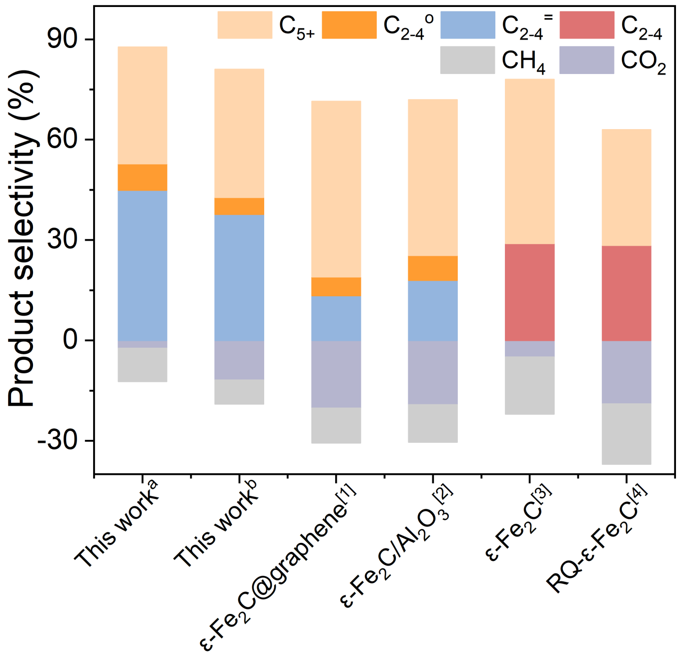

Supplementary Fig. 12 | Comparison of the catalytic performance of ε-Fe_2C-0.4Mn with that of other previously reported ε-Fe_2C catalysts. (a: reaction conditions: 0.10 g of catalyst, 280 °C, 2.0 MPa, H_2/CO = 2.5, GHSV = 60 L·g_{cat}^{-1}·h^{-1}, b: reaction conditions: 0.10 g of catalyst, 300 °C, 2.0 MPa, H_2/CO = 2.5, GHSV = 20 L·g_{cat}^{-1}·h^{-1})

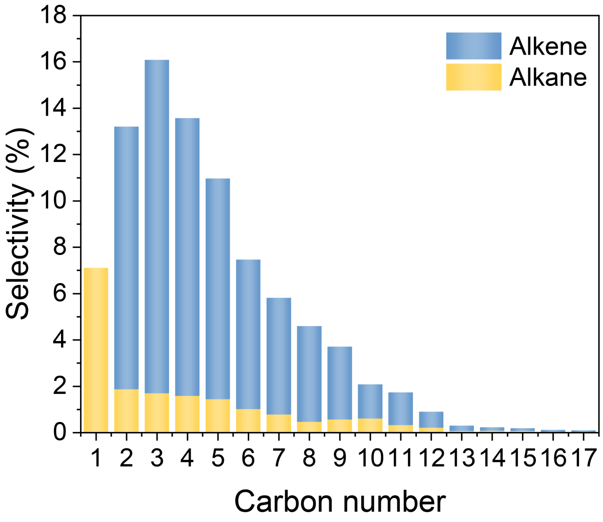

Supplementary Fig. 13 | Product distribution of ε-Fe_2C-Mn catalyst. Detailed product selectivity over ε-Fe_2C-Mn catalyst. Reaction conditions: WHSV: 20000 h^{-1}, H_2/CO = 2.5:1, 300 °C, 2 MPa. In the product distribution of ε-Fe_2C-Mn catalyst, it can be seen that the alkene is dominated in the product. The products were mainly distributed below C12. This product distribution suggests that the ε-Fe_2C-Mn catalyst is very suitable for the production of unsaturated hydrocarbons.

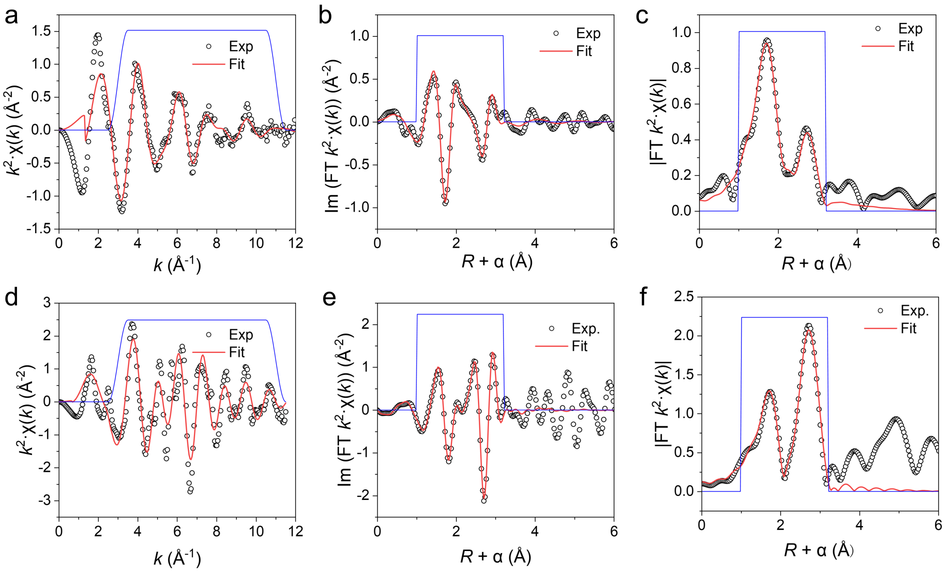

Supplementary Fig. 14 | Curve-fit (red line) of Mn *K*-edge EXAFS (black dot). ε-Fe_2C-0.06Mn: (a) *k*-space, (b) *R*-space imaginary part and (c) *R*-space; ε-Fe_2C-0.4Mn: (d) *k*-space, (e) *R*-space imaginary part and (f) *R*-space. The blue line is the fitting windows. The data are k^2-weighted and without phase correction.

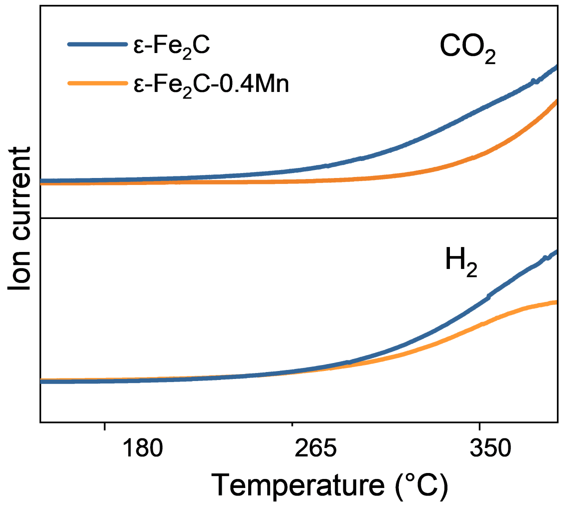

Supplementary Fig. 15 | Temperature Programmed Surface Reaction (TPSR) experiment of $CO+H_2O=CO_2+H_2$. The signal of CO_2 and H_2 was detected by the mass spectrometer.

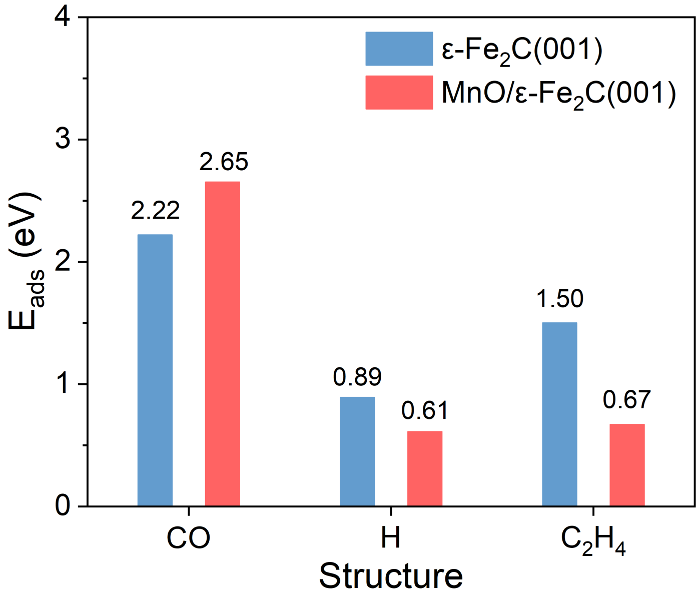

Supplementary Fig. 16 | The adsorption energies of CO, H, and C_2H_4 at their optimum adsorption configurations on ε-Fe_2C (001) and MnO/ε-Fe_2C (001).

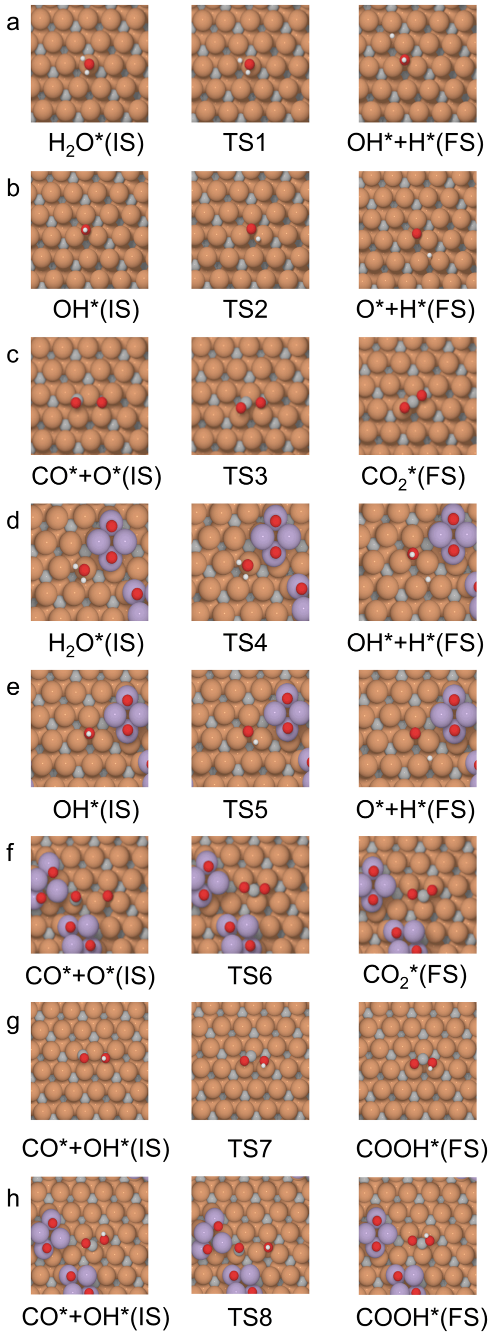

Supplementary Fig. 17 | The corresponding structures of H_2O dissociation and CO_2 formation on ε-Fe_2C (001) and MnO/ε-Fe_2C (001). (a) Initial, transition, and final states of H_2O dissociation on ε-Fe_2C (001). (b) Initial, transition, and final states of OH dissociation on ε-Fe_2C (001). (c) Initial, transition, and final states of CO reacting with O on ε-Fe_2C (001). (d) Initial, transition, and final states of H_2O dissociation on MnO/ε-Fe_2C (001). (e) Initial, transition, and final states of OH dissociation on MnO/ε-Fe_2C (001). (f) Initial, transition, and final states of CO reacting with O on MnO/ε-Fe_2C (001). (g) Initial, transition, and final states of CO reacting with OH on ε-Fe_2C (001). (h) Initial, transition, and final states of CO reacting with OH on MnO/ε-Fe_2C (001). (Fe: brown; C: grey; O: red; Mn: purple; H: white).

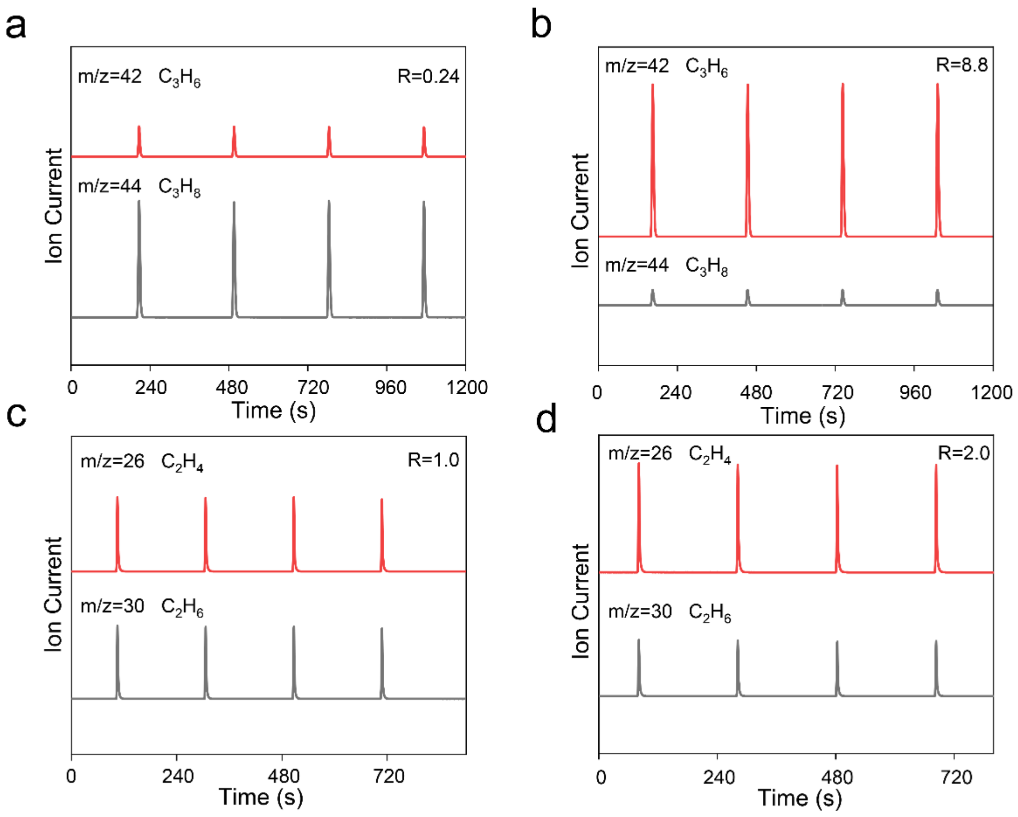

Supplementary Fig. 18 | C_3H_6 and C_2H_4 hydrogenation experiment. (a) C_3H_6 hydrogenation in ε-Fe_2C **(b)** C_3H_6 hydrogenation in ε-Fe_2C-0.4Mn **(c)** C_2H_4 hydrogenation in ε-Fe_2C **(d)** C_2H_4 hydrogenation in ε-Fe_2C-0.4Mn. At 573 K, 100 μL pure C_3H_6 or C_2H_4 is pulsed into H_2 (50 mL·min^{-1}) to obtain a transient response curve of ε-Fe_2C and ε-Fe_2C-0.4Mn. R is the integrated peak area ratio of C_3H_6/C_3H_8 or C_2H_4/C_2H_6 detected by the mass spectrometer.

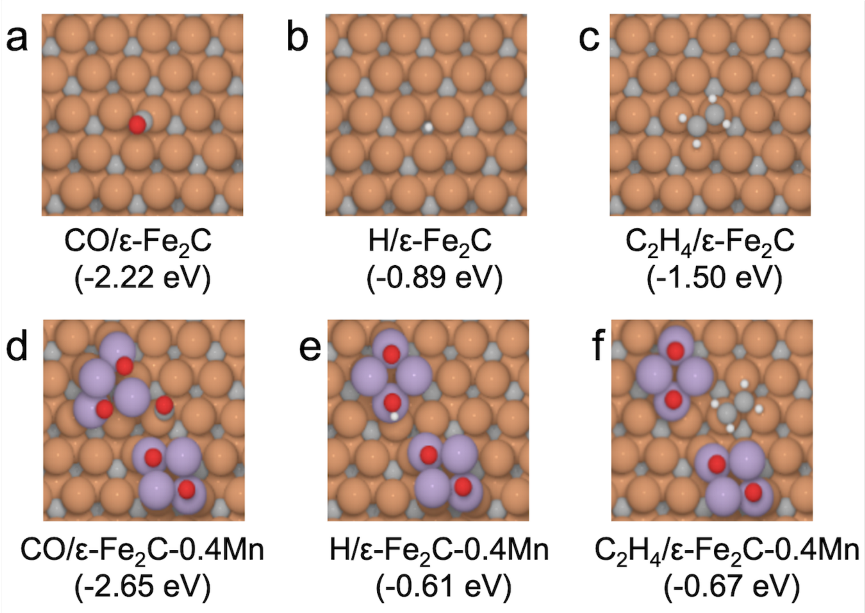

Supplementary Fig. 19 | Optimal configurations and adsorption energies of CO, H, and C_2H4 molecules on ε-Fe_2C (001) and MnO/ε-Fe_2C (001), respectively. (a) CO on ε-Fe_2C (001), (b) H on ε-Fe_2C (001), (c) C_2H_4 on ε-Fe_2C (001), (d) CO on MnO/ε-Fe_2C (001), (e) H on MnO/ε-Fe_2C (001), (f) C_2H_4 on MnO/ε-Fe_2C (001). (Fe: brown; C: grey; O: red; Mn: purple; H: white).

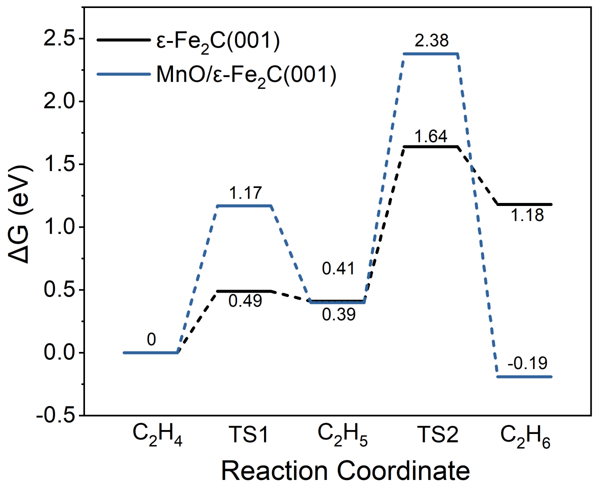

Supplementary Fig. 20 | The free energy profile for propene hydrogenation into propane on ε-Fe_2C (001) and MnO/ε-Fe_2C (001), respectively.

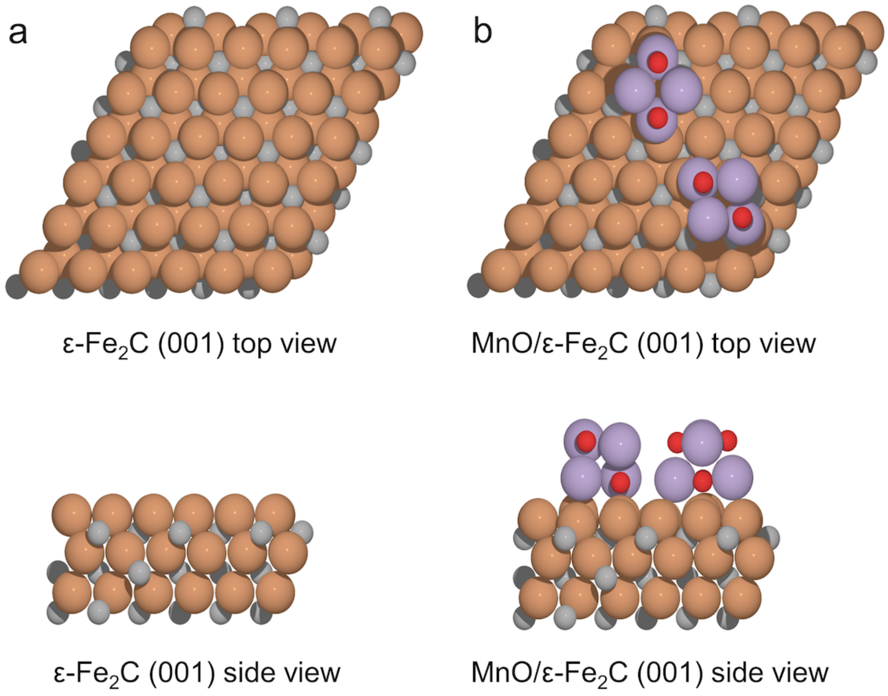

Supplementary Fig. 21 | Computational models of ab initio nanoreactor simulations and static DFT calculations for bare ε-Fe_2C (001) and MnO/ε-Fe_2C (001). (a) Theoretical model of ε-Fe_2C (001) (b) Theoretical model of MnO/ε-Fe_2C (001)

Supplementary Table 1 | Fitting parameters of the Operando Mössbauer spectra of ε-Fe_2C-0.4Mn catalyst. (corresponding to Supplementary Fig. 3)

Sample	IS ($mm \cdot s^{-1}$)	QS ($mm \cdot s^{-1}$)	Hhf (kOe)	Γ/2 ($mm \cdot s^{-1}$)	Area (%)	Assignment
ε-Fe_2C-0.4Mn-2h	0.34	0.01	15.94	0.84	96.68	Fe_2C
	0.33	0.89	-	0.29	3.32	Fe^{3+}
ε-Fe_2C-0.4Mn-4h	0.33	0.00	16.11	0.77	96.57	Fe_2C
	0.32	0.90	-	0.29	3.43	Fe^{3+}
ε-Fe_2C-0.4Mn-6h	0.32	0.00	16.27	0.79	97.03	Fe_2C
	0.32	0.88	-	0.29	2.97	Fe^{3+}

Supplementary Table 2 | Fitting parameters of the Mössbauer spectra of ε-Fe_2C catalyst under different activation conditions. (corresponding to Supplementary Fig. 4b)

Sample	IS ($mm \cdot s^{-1}$)	QS ($mm \cdot s^{-1}$)	Hhf (kOe)	Γ/2 ($mm \cdot s^{-1}$)	Area (%)	Assignment
NH_3	0.24	0.08	23.34	0.84	97.13	Fe_2C
	0.22	1.16	-	0.29	2.87	Fe^{3+}
5%CO-He	0.15	0.01	16.19	0.58	84.02	Fe_2C
	0.18	1.00	-	0.29	2.85	Fe^{3+}
	0.40	0.44	48.08	0.29	2.50	Fe_3O_4 (A)
	0.50	0.27	44.00	0.64	10.63	Fe_3O_4 (B)
He	0.31	0.04	16.83	0.56	79.01	Fe_2C
	0.30	0.88	-	0.29	2.06	Fe^{3+}
	0	-0.98	47.50	0.29	1.63	Fe_3O_4 (A)
	0.5	0.09	44.00	0.92	17.30	Fe_3O_4 (B)

Supplementary Table 3 | Catalytic performance of ε-Fe_2C catalyst under different activation conditions. (corresponding to Supplementary Fig. 3c)

Activation conditions	CO Conversion [%]	C_1 sel.[mol%]			C_{2-4} sel. [mol%]	C_{5+}sel. [mol%]	O/P
		CO_2	CH_4	Total			
NH_3	67	34	21	55	27	18	1.1
5%CO-He	48	30	20	50	25	23	0.88
He	40	32	20	52	25	25	0.77

Reaction conditions: 0.10 g catalyst, 270 °C, GHSV = 60 $L\cdot g_{cat}^{-1}\cdot h^{-1}$, 2 MPa and H_2/CO ratio of 2.

Supplementary Table 4 | The ratio of N/Fe from X-ray energy dispersive spectroscopy (EDS). (corresponding to Supplementary Fig. 4)

Sample	ε-Fe_2C-spent	ε-Fe_2C-0.06Mn-spent	ε-Fe_2C-0.4Mn-spent
Ratio of N/Fe (%)	2.2	2.5	2.6

Supplementary Table 5 | Fitting parameters of the Mössbauer spectra of the ε-Fe_2C catalysts before reaction with different manganese additions. (corresponding to Supplementary Fig. 7)

Sample	IS ($mm \cdot s^{-1}$)	QS ($mm \cdot s^{-1}$)	Hhf (kOe)	Γ/2 ($mm \cdot s^{-1}$)	Area (%)	Assignment
ε-Fe_2C- fresh	0.1	0.2	-	0.9	100	Fe_2N
ε-Fe_2C-0.06Mn-fresh	0.1	0.2	-	0.9	100	Fe_2N
ε-Fe_2C-0.4Mn- fresh	0.1	0.2	-	0.9	100	Fe_2N

Supplementary Table 6 | Fitting parameters of the Mössbauer spectra of the ε-Fe_2C catalysts with different manganese additions. (corresponding to Fig. 2 f)

Sample	IS ($mm{\cdot}s^{-1}$)	QS ($mm{\cdot}s^{-1}$)	Hhf (kOe)	Γ/2 ($mm{\cdot}s^{-1}$)	Area (%)	Assignment
ε-Fe_2C	0.25	0.05	17.96	0.64	96.03	Fe_2C
	0.26	-0.93	-	0.29	3.97	Fe^{3+}
ε-Fe_2C-0.06Mn	0.25	0.05	17.81	0.60	95.76	Fe_2C
	0.26	-0.93	-	0.29	4.24	Fe^{3+}
ε-Fe_2C-0.4Mn	0.26	0.02	17.81	0.61	92.10	Fe_2C
	0.25	-1.00	-	0.58	7.90	Fe^{3+}

Supplementary Table 7 | Catalytic properties of ε-Fe_2C with different manganese additions level.

Catalysts	CO Conversion [%]	C_1 sel.[mol%]			C_{2-4} sel. [mol%]	C_{5+}sel. [mol%]	O/P	Hydrocarbon (Excluded CO_2)				Carbon balance [%]
		CO_2	CH_4	Total				CH_4	C_{2-4}	C_5^+	$C_{2+}^=$	
ε-Fe_2C^a	42.8	32.7	25.4	58.1	24.7	17.2	0.9	37.7	36.7	25.6	24.3	102.0
ε-Fe_2C-0.02Mn^b	42.0	29.8	17.2	50.2	26.8	23.0	2.3	25.7	40.0	34.3	46.8	101.1
ε-Fe_2C-0.04Mn^c	41.1	22.4	11.1	33.5	29.0	37.5	4.5	14.3	37.4	48.3	63.1	98.2
ε-Fe_2C-0.06Mn^d	43.0	17.6	8.0	25.6	30.0	44.4	7.1	9.7	36.4	53.9	74.9	97.8
ε-Fe_2C-0.2Mn^e	42.1	17.0	7.5	24.5	32.6	42.9	7.2	9.0	39.3	51.7	76.2	97.1
ε-Fe_2C-0.4Mn^f	41.0	11.9	7.1	19.0	42.8	38.2	7.4	8.1	48.6	43.4	79.7	96.8
ε-Fe_2C-0.6Mn^g	40.3	14.8	8.9	23.7	48.1	28.2	6.4	10.4	56.5	33.1	75.8	98.6

Reaction conditions: 0.10 g catalyst, 300 °C, 2.0 MPa, $H_2/CO = 2.5$, gas hourly space velocity (GHSV) varied from 20 to 400 $L \cdot g_{cat}^{-1} \cdot h^{-1}$ to obtain CO conversion of 41.5 ± 1.5%.

Supplementary Table 8 | Catalytic properties of ε-Fe_2C-0.4Mn under different ratio of H_2/CO.

Ratio of H_2/CO	CO Conversion [%]	C_1 sel.[mol%]			C_{2-4} sel. [mol%]	C_{5+}sel. [mol%]	O/P	Hydrocarbon (Excluded CO_2)				Carbon balance [%]
		CO_2	CH_4	Total				CH_4	C_{2-4}	C_5^+	$C_{2+}^=$	
0.5	31.8	33.4	4.2	37.6	20.6	41.8	8.4	6.3	30.9	62.8	78.5	101.7
1	33.6	22.6	3.7	26.3	26.0	47.7	9.0	4.8	33.6	61.6	81.5	99.3
1.5	37.8	17.9	5.2	23.1	32.6	44.3	8.2	6.4	39.7	53.9	80.0	97.5
2	39.8	14.0	6.5	20.5	38.5	41.0	7.7	7.6	44.8	47.7	80.0	96.9
2.5	41.0	11.9	7.1	19.0	42.8	38.2	7.4	8.1	48.6	43.3	79.7	96.8
3	42.8	9.63	10.2	19.8	48.1	32.1	6.4	11.3	53.2	35.5	75.5	97.2

Reaction conditions: 0.10 g catalyst, GHSV = $20L·g_{cat}^{-1}·h^{-1}$, 2 MPa and temperature of 300 °C.

Supplementary Table 9 | Catalytic properties of ε-Fe_2C-0.4Mn under different pressure.

Pressure (MPa)	CO Conversion [%]	C_1 sel.[mol%]			C_{2-4} sel. [mol%]	C_{5+}sel. [mol%]	O/P	Hydrocarbon (Excluded CO_2)				Carbon balance [%]
		CO_2	CH_4	Total				CH_4	C_{2-4}	C_5^+	$C_{2+}^=$	
0.5	26.4	17.9	18.5	36.4	43.2	20.4	6.65	22.5	52.6	24.8	66.3	103.2
1	27.5	9.64	9.0	18.6	43.5	37.9	7.37	10.0	48.1	41.9	76.9	97.6
1.5	32.1	10.0	6.8	16.8	44.0	39.2	7.00	7.6	48.9	43.5	78.5	99.2
2	41.0	11.9	7.1	19.0	42.8	38.2	7.35	8.1	48.6	43.3	79.7	96.8
2.5	42.0	14.0	7.7	21.7	42.1	36.2	6.04	9.0	49.0	42.1	75.8	97.7

Reaction conditions: 0.10 g catalyst, GHSV = 20 $L \cdot g_{cat}^{-1} \cdot h^{-1}$, 300 °C and H_2/CO ratio of 2.5.

Supplementary Table 10 | Catalytic properties of ε-Fe_2C-0.4Mn under different temperature.

Temperature (℃)	CO Conversion [%]	C_1 sel.[mol%]			C_{2-4} sel. [mol%]	C_5^+sel. [mol%]	O/P	Hydrocarbon (Excluded CO_2)				Carbon balance [%]
		CO_2	CH_4	Total				CH_4	C_{2-4}	C_5^+	$C_{2+}^=$	
280	25.7	9.00	6.66	15.7	47.0	37.3	6.41	7.32	51.6	41.0	77.1	97.1
290	30.2	9.17	7.68	16.9	47.5	35.7	6.50	8.46	52.3	39.2	76.6	97.9
300	41.0	11.9	7.14	19.0	42.8	38.2	7.35	8.10	48.6	43.3	79.7	96.8
320	52.0	15.2	8.17	23.4	38.8	37.8	6.71	9.63	45.8	44.6	75.5	98.3
340	71.9	21.5	15.7	37.2	38.3	24.5	4.22	20.0	48.8	31.2	64.6	102.1

Reaction conditions: 0.10 g catalyst, GHSV= 20 $L \cdot g_{cat}^{-1} \cdot h^{-1}$, 2 MPa and H_2/CO ratio of 2.5.

Supplementary Table 11 | Comparison of the performance of ε-Fe_2C-0.4Mn with Mn-promoted Fe-based catalysts reported in the literature.

Entry	Catalysts	WHSV ($ml\cdot g_{cat}^{-1}\cdot h^{-1}$)	T (°C)	H_2/CO ratio	CO Conv [%]	Sel. [mol%]						Ref.
						CO_2	CH_4	C1 Total	C_{2-4}	C_{5+}	Olefins	
1	Mn/γ-Fe_2O_3	4480	320	1	57.1	31.7	8.0	39.7	47.9	12.4	41.8[a]	(5)
2	Fe-MnK-AC	3000	320	1	85.0	48	11.8	59.8	24.8	15.4	20.5[a]	(6)
3	FeMnLi	5000	320	2	85.6	34.6	9.3	43.9	32.5	23.6	24.0[a]	(7)
4	Fe_3O_4@MnO_2	11000	340	2	91.8	37.9	7.5	45.4	26.2	28.4	23.2[a]	(8)
5	FeMnCu	1500	300	2	96.9	23.0	15.4	38.4	54.3	7.3	30.9[a]	(9)
6	Fe-Mn(4:1)	1500	260	1	5.5	20.7	15.0	35.7	53.8	10.5	38.6[a]	(10)
7	Mn/Fe_3O_4	4480	320	1	41.5	37.8	6.0	43.8	41.5	14.7	37.4[a]	(11)
8	$Mn_xFe_{3-x}O_4$	4000	260	1	7.1	25.2	12.7	37.9	52.1	10.0	40.7[a]	(12)
9	Fe_4Mn_1	7500	280	1	32.4	42.8	11.3	54.1	37.3	8.6	29.3[a]	(13)
10	100Fe7Mn	2000	250	2	45.1	19.2	9.6	28.8	35.3	35.9	26.8[a]	(14)
11	Mn-KCuFe/mAl_2O_3	2000	270	1.25	95.0	39.4	6.1	45.5	5.5	49.0	4.7[a]	(15)
12	FeMnCu/MCF-0	6000	270	1	40.9	11.7	18.4	30.1	44.8	25.1	28.8[a]	(16)
13	Fe@12.42Mn	4000	265	2	42.0	14.4	8.4	22.8	36.2	41.0	28.7[a]	(17)
14	$Fe_{2.86}Mn_{0.14}O_4$/CNT	6000	300	1	43.9	37.2	3.8	41.0	25.2	33.8	19.8[a]	(18)
15	ε-Fe_2C-0.4Mn	20000	300	2.5	41.0	11.9	7.1	19.0	42.8	38.2	70.2	This work
16	ε-Fe_2C-0.4Mn	60000	280	2.5	17.1	2.4	9.9	12.3	52.8	34.9	71.3	This work

a: The values denote the selectivity of lower olefins ($C_{2-4}^{=}$)

Supplementary Table 12 | Comparison of the catalytic performance of ε-Fe_2C-Mn with syngas-to-olefins systems and other Fe_2C catalysts reported in the literature.

Entry	Catalysts	WHSV ($ml \cdot g_{cat}^{-1} \cdot h^{-1}$)	T (°C)	H_2/CO ratio	CO Conv [%]	Sel. [mol%]						Ref.
						CO_2	CH_4	C1 Total	C_{2-4}	C_{5+}	Olefins	
1	ZnCrOx-MSAPO	5143	400	2.5	17.0	45.0	1.2	46.2	39.4	5.4	47.2[a]	(19)
2	CoMnC/PDVB	1800	250		63.5	46.3	2.6	48.9	39.8	11.3	38.3[a]	(20)
3	0.5Na/CoMnAl@6.6Si	4000	260	0.5	13.5	16.7	4.3	21.0	36.9	42.1	61.1	(21)
4	CoMn	2000	250	2	31.8	47.3	2.6	49.9	33.1	17.0	60.8[a]	(22)
5	Co_1Mn_3-Na_2S	-	240	2	0.8	< 3	17	< 20	-	-	54.0	(23)
6	Na-FeCx/s-ZSM-5	2400	260	1	82.5	46.6	1.6	48.2	20.3	30.8	38.4[a]	(24)
7	Fe/α-Al_2O_3	1500	340	1	80.0	40	6.6	46.6	-	-	31.8[a]	(25)
8	FeMn@Si-c	4000	320	2	56.1	13.0	10.0	23.0	-	-	64.9	(26)
9	Fe-Zn-0.81Na	60000	340	2.7	77.2	23.8	9.7	33.5	25.9	40.6	52.5	(27)
10	Fe_3O_4@MnO_2	3000	280	1	67.9	47.1	3.6	50.7	-	-	41.9	(28)
11	ε-Fe_2C@graphene	-	300	1	-	20.3	8.2	28.5	19.0	52.4	13.4[a]	(1)
12	ε-Fe_2C/Al_2O_3	9000	280	1	-	19.2	9.0	28.2	25.3	46.5	18.0[a]	(2)
13	ε-Fe_2C	18000	235	1.5	15.0	5.0	17.0	22.0	29.0	49.0	-	(3)
14	RQ-ε-Fe_2C	-	170	2	76.0	19.0	18.0	37.0	28.4	34.6	-	(4)
15	ε-Fe_2C-0.4Mn	20000	300	2.5	41.0	11.9	7.1	19.0	42.8	38.2	70.2	This work
16	ε-Fe_2C-0.4Mn	60000	280	2.5	17.1	2.4	9.9	12.3	52.8	34.9	71.3	This work

a: The values denote the selectivity of lower olefins ($C_{2-4}^{=}$)

Supplementary Table 13 | Curve-fit Parameters [a] for Mn *K*-edge EXAFS of ε-Fe_2C-xMn.

Catalyst	Path	*d* (Å) [b]	C. N. [c]	ΔE_0 (eV)	σ^2 (Å^2) [d]	*R*-factor
ε-Fe_2C-0.06Mn	Mn-O	2.14 ± 0.01	4.1 ± 0.5	7 ± 1	0.009 ± 0.003	0.016
	Mn-Mn(Fe)	3.15 ± 0.02	2.3 ± 1.3		0.012 ± 0.007	
ε-Fe_2C-0.4Mn	Mn-O	2.19 ± 0.01	5.8 ± 1.0	2 ± 1	0.007 ± 0.003	0.009
	Mn-Mn(Fe)	3.14 ± 0.01	12.1 ± 1.8		0.010 ± 0.002	

a: The data ranges used in these fittings are $3.0 \leq k \leq 11.0$ Å-1 and $1.0 \leq R \leq 3.2$ Å. S02 was fixed at 0.829, obtained from the MnO powder measured at the same time. The number of variable parameters in each fitting is out of total of 11.0 independent data point.

b: The coordination distance. The distances for Mn-O and Mn-Mn are from the crystal structure of MnO (Fm-3m, ICSD collection code 9864).

c: Average coordination number.

d: Debye-Waller factor.

Supplementary Table 14 | Catalytic properties of ε-Fe_2C mechanical mixed MnO.

Catalysts	CO Conversion [%]	C_1 sel.[mol%]			C_{2-4} sel. [mol%]	C_{5+}sel. [mol%]	O/P
		CO_2	CH_4	Total			
ε-Fe_2C-0Mn	98.1	35.8	30.1	65.9	26.1	8.0	0.9
ε-Fe_2C#0.4Mn[a]	96.5	33.4	25.2	58.6	32.1	9.3	0.8
ε-Fe_2C#0.4Mn[b]	96.3	34.6	24.2	58.8	32.0	9.2	0.9
ε-Fe_2C-0.4Mn	41.0	11.9	7.1	19.0	42.9	38.2	7.4

Reaction conditions: 0.10 g catalyst, 300 °C, GHSV= 20 $L\cdot g_{cat}^{-1}\cdot h^{-1}$, 2 MPa and H_2/CO ratio of 2.5.

a: The fresh catalysts and commercial MnO were mechanically mixed and then pelleted.

b: The fresh catalysts and commercial MnO were pelleted and then mechanically mixed.

Supplementary References

1. Xu, K. et al. ε-Iron carbide as a low-temperature Fischer–Tropsch synthesis catalyst. *Nat. Commun.* **5**, 5783 (2014).
2. Fu, X. et al. Supported Fe_2C catalysts originated from Fe_2N phase and active for Fischer-Tropsch synthesis. *Appl. Catal. B: Environ.* **284**, 119702 (2021).
3. Wang, P. et al. Synthesis of stable and low-CO_2 selective epsilon-iron carbide Fischer-Tropsch catalysts. *Sci. Adv.* **4**, eaau2947 (2018).
4. Lyu, S. et al. Stabilization of epsilon-iron carbide as high-temperature catalyst under realistic Fischer-Tropsch synthesis conditions. *Nat. Commun.* **11**, 6219 (2020).
5. Liu, Y. et al. Effects of initial crystal structure of Fe_2O_3 and Mn promoter on effective active phase for syngas to light olefins. *Appl. Catal. B: Environ.* **261**, 118219 (2020).
6. Tian, Z. et al. Fischer-Tropsch synthesis to light olefins over iron-based catalysts supported on $KMnO_4$ modified activated carbon by a facile method. *Appl. Catal. A: Gen.* **541**, 50-59 (2017).
7. Wu, X. et al. Li-decorated Fe-Mn nanocatalyst for high-temperature Fischer–Tropsch synthesis of light olefins. *Fuel* **257**, 116101 (2019).
8. Wu, X. et al. High-temperature Fischer–Tropsch synthesis of light olefins over nano-Fe_3O_4@MnO_2 core–shell catalysts. *Ind. Eng. Chem. Res.* **58**, 21350-21362 (2019).
9. Gong, W. et al. Effect of copper on highly effective Fe-Mn based catalysts during production of light olefins via Fischer-Tropsch process with low CO_2 emission. *Appl. Catal. B: Environ.* **278**, 120683 (2020).
10. Yang, Z. et al. Tuning direct CO hydrogenation reaction over Fe-Mn bimetallic catalysts toward light olefins: Effects of Mn promotion. *Appl. Catal. B: Environ.* **285**, 119815 (2021).
11. Liu, Y., Chen, J., Bao, J., Zhang, Y. Manganese-Modified Fe_3O_4 microsphere catalyst with effective active phase of forming light olefins from syngas. *ACS Catal.* **5**, 3905-3909 (2015).
12. Shi, B. et al. Promotional effect of Mn-doping on the structure and performance of spinel ferrite microspheres for CO hydrogenation. *J. Catal.* **381**, 150-162 (2020).
13. Ding, X., Zhu, M., Han, Y., Yang, Z. Revisiting the syngas conversion to olefins over Fe-Mn bimetallic catalysts: Insights from the proximity effects. *J. Catal.* **417**, 213-225 (2023).
14. Li, T., Wang, H., Yang, Y., Xiang, H., Li, Y. Effect of manganese on the catalytic performance of an iron-manganese bimetallic catalyst for light olefin synthesis. *J. Energy Chem.* **22**, 624-632 (2013).
15. Badoga, S., Kamath, G., Dalai, A. Effects of promoters (Mn, Mg, Co and Ni) on the Fischer-Tropsch activity and selectivity of KCuFe/mesoporous-alumina catalyst. *Appl. Catal. A: Gen.* **607**, 117861 (2020).
16. Huang, S. et al. The effect of Mn on the performance of MCF-supported highly dispersed iron catalysts for Fischer–Tropsch synthesis. *Catal. Sci. Technol.* **10**, 502-509 (2020).
17. Wang, C., Zhang, T., Zhu, H., Zhang, J., Chen, J. Exploring the promotion behavior of manganese in Fischer–Tropsch synthesis by changing the interaction patterns between manganese and iron. *Fuel* **360**, 130567 (2024).
18. Xu, J. et al. Carbon nanotube-supported Fe–Mn nanoparticles: A model catalyst for direct conversion of syngas to lower olefins. *Catal. Today* **215**, 86-94 (2013).
19. Jiao, F. et al. Selective conversion of syngas to light olefins. *Science* **351**, 1065-1068 (2016).

20. Fang, W. et al. Physical mixing of a catalyst and a hydrophobic polymer promotes CO hydrogenation through dehydration. *Science* **377**, 406-410 (2022).
21. Lin, T. et al. Designing silica-coated CoMn-based catalyst for Fischer-Tropsch synthesis to olefins with low CO_2 emission. *Appl. Catal. B: Environ.* **299**, 120683 (2021).
22. Zhong, L. et al. Cobalt carbide nanoprisms for direct production of lower olefins from syngas. *Nature* **538**, 84-87 (2016).
23. Xie, J. et al. Promoted cobalt metal catalysts suitable for the production of lower olefins from natural gas. *Nat. Commun.* **10**, 167 (2019).
24. Wang, C. et al. Fischer-Tropsch synthesis to olefins boosted by MFI zeolite nanosheets. *Nat. Nanotechnol.* **17**, 714-720 (2022).
25. Torres Galvis, H. M. et al. Supported iron nanoparticles as catalysts for sustainable production of lower olefins. *Science* **335**, 835-838 (2012).
26. Xu, Y. et al. A hydrophobic FeMn@Si catalyst increases olefins from syngas by suppressing C1 by-products. *Science* **371**, 610-613 (2021).
27. Zhai, P. et al. Highly tunable selectivity for syngas-derived alkenes over zinc and sodium-modulated Fe_5C_2 catalyst. *Angew. Chem. Int. Ed.* **55**, 9902-9907 (2016).
28. Wang, J. et al. Directly converting syngas to linear α-olefins over core–shell Fe_3O_4@MnO_2 Catalysts. *ACS Appl. Mater. Interfaces.* **10**, 43578-43587 (2018).
